# ROS-Responsive 4D Printable Acrylic Thioether-Based Hydrogels for Smart Drug Release

**DOI:** 10.1021/acs.chemmater.3c02264

**Published:** 2023-12-13

**Authors:** Maria Regato-Herbella, Isabel Morhenn, Daniele Mantione, Giuseppe Pascuzzi, Antonela Gallastegui, Ana Beatriz Caribé dos Santos Valle, Sergio E. Moya, Miryam Criado-Gonzalez, David Mecerreyes

**Affiliations:** † POLYMAT University of the Basque Country UPV/EHU, Joxe Mari Korta Center. Avda. Tolosa 72, 20018 Donostia-San Sebastián, Spain; ‡ Center for Cooperative Research in Biomaterials (CIC biomaGUNE), Basque Research and Technology Alliance (BRTA), Paseo de Miramón 194, 20014Donostia-San Sebastián, Spain; § Ikerbasque, Basque Foundation for Science, 48013 Bilbao, Spain; ∥ Department of Chemistry, Materials and Chemical Engineering “Giulio Natta”, Politecnico di Milano, Piazza Leonardo da Vinci 32, 20133 Milano ,Italy

## Abstract

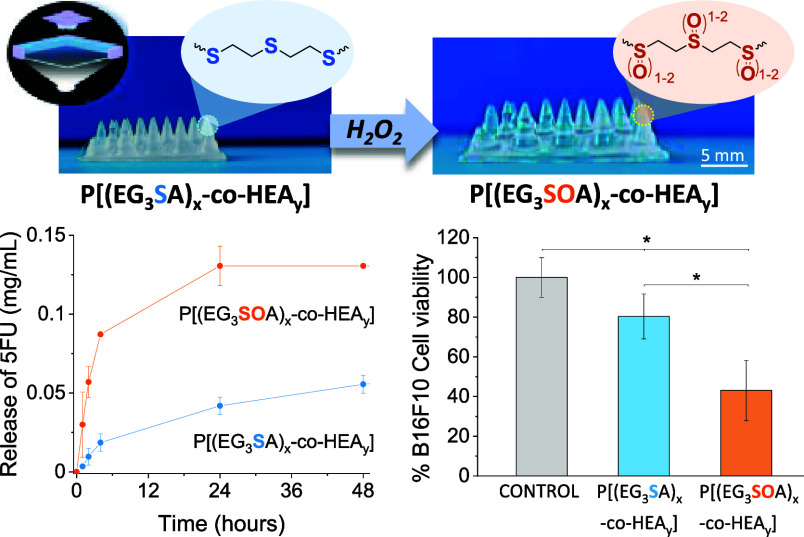

Reactive oxygen species (ROS) play a key role in several biological functions like regulating cell survival and signaling; however, their effect can range from beneficial to nondesirable oxidative stress when they are overproduced causing inflammation or cancer diseases. Thus, the design of tailor-made ROS-responsive polymers offers the possibility of engineering hydrogels for target therapies. In this work, we developed thioether-based ROS-responsive difunctional monomers from ethylene glycol/thioether acrylate (EG_*n*_SA) with different lengths of the EG_*n*_ chain (*n* = 1, 2, 3) by the thiol-Michael addition click reaction. The presence of acrylate groups allowed their photopolymerization by UV light, while the thioether groups conferred ROS-responsive properties. As a result, smart PEG_*n*_SA hydrogels were obtained, which could be processed by four-dimensional (4D) printing. The mechanical properties of the hydrogels were determined by rheology, pointing out a decrease of the elastic modulus (*G*′) with the length of the EG segment. To enhance the stability of the hydrogels after swelling, the EG_*n*_SA monomers were copolymerized with a polar monomer, 2-hydroxyethyl acrylate (HEA), leading to P[(EG_*n*_SA)_*x*_-*co*-HEA_*y*_] with improved compatibility in aqueous media, making it a less brittle material. Swelling properties of the hydrogels increased in the presence of hydrogen peroxide, a kind of ROS, reaching values of ≈130% for P[(EG_3_SA)_7_-*co*-HEA_93_] which confirms the stimuli-responsive properties. Then, the P[(EG_3_SA)_*x*_-*co*-HEA_*y*_] hydrogels were employed as matrixes for the encapsulation of a chemotherapeutic drug, 5-fluorouracil (5FU), which showed sustained release over time modulated by the presence of H_2_O_2_. Finally, the effect of the 5-FU release from P[(EG_3_SA)_*x*_-*co*-HEA_*y*_] hydrogels was tested *in vitro* with melanoma cancer cells B16F10, pointing out B16F10 growth inhibition values in the range of 40–60% modulated by the EG_3_SA percentage and the presence or absence of ROS agents, thus confirming their excellent ROS-responsive properties for the treatment of localized pathologies.

## 1 Introduction

Hydrogels, three-dimensional networks with the ability to hold a large quantity of water, have been widely employed in the biomedical field in applications such as drug delivery,^1,2^ tissue engineering,^3,4^ or biosensing.^5,6^ The design of tailor-made stimuli-responsive polymers offers the possibility of engineering smart hydrogels with desired biodegradability, biocompatibility, and mechanical strength that can be processed by advanced additive manufacturing technologies.^7−10^ The ability of hydrogels to change their properties over time upon response to specific biological stimuli, i.e., temperature,^11^ pH,^12^ enzyme activity,^13^ or redox balance,^14,15^ make them ideal candidates for four-dimensional (4D) printing. 4D printing is an emerging processing technology with growing interest in the fabrication of dynamic shape-defined materials capable of easily adapting to different environments and applications beyond conventional materials and technologies.^16,17^

Reactive oxygen species (ROS) that are oxidant species present in the human body, i.e., hydrogen peroxide (H_2_O_2_), play a pivotal role in several biological functions.^18,19^ ROS effects can range from beneficial cell survival and signaling to nondesirable oxidative stress when they are overproduced, causing inflammation, cancer, and age-related diseases.^20,21^ Thus, the development of ROS-sensitive polymer materials with defined structures that can control the ROS concentration is actively searched. There exist different types of ROS-responsive polymers depending on the ROS active unit, i.e., sulfides, diselenides, thioketals, aryl boronic esters, and so forth.^22^ Among them, those bearing thioether groups have interesting properties resulting from their ability to be oxidized in the presence of ROS experiencing a hydrophobic to hydrophilic transition without the need to be cleaved.^23^ Different chemical strategies can be employed to synthesize ROS-response thioether-based polymers in which the thioether group can be located in the main, side, or tail chains. As examples of the thioether group present in the main chain, we can mention the amphiphilic triblock copolymers made of hydrophilic poly(ethylene glycol) (PEG) and hydrophobic poly(propylene sulfide) (PPS), PEG-*b*-PPS-*b*-PEG, synthesized by Hubbell and co-workers.^24^ In this pioneering work, the authors demonstrated the transformation of hydrophobic thioether groups into hydrophilic sulfoxide or sulfone groups when the polymer was oxidized in the presence of H_2_O_2_ or hypochlorite, respectively. Besides, many amphiphilic block copolymers formed by PEG as the hydrophilic segment and different hydrophobic polymers such as polystyrene (PS), PEG-*b*-PS, poly(ε-caprolactone) (PCL), PEG-*b*-PCL, or poly(β-thioether ester) (PTE), PEG-*b*-PTE, have been synthesized.^25,26^ Their amphiphilic nature makes them suitable for the fabrication of nano- and microparticles through their self-assembly in aqueous media, whereas it makes difficult their green processability in the form of hydrogels.^27−31^ In this later case, there are few examples focused on the synthesis of thioether-based hydrogels, which are related to the incorporation of amino acids such as l-methionine, cysteine, and polyserine in the polymer chain leading to thioether-based polypeptides macrogels without defined morphological structures, which could be employed as ROS scavengers in redox microenvironments.^32−35^ From the functional point of view,^36^ high-definition complex structures are of great interest in reproducing key features of the cellular microenvironment favoring cell-facing constructs to engineer implantable microscaffolds and organ-on-a-chip devices.^37^ To that aim, digital light printing (DLP) attracts great attention as it allows to fabricate high-resolution structures not achievable with conventional printing techniques, which makes it necessary to develop photopolymerizable inks.^38^ In this regard, to the best of our knowledge, the synthesis of hydrophilic and photopolymerizable thioether-based ROS-responsive polymers for the fabrication of high-resolution 4D printable hydrogels has not been previously reported. We present here the synthesis of new aqueous soluble redox monomers from ethylene glycol sulfur acrylate (EG_*n*_SA) with different lengths of the EG_*n*_ chain (*n* = 1, 2, 3), which can be photopolymerized by UV light leading to hydrogels. The resulting hydrogels are fully characterized to determine their physical and chemical properties, including ROS responsivity. The processing of the PEG_*n*_SA hydrogels by digital light 4D printing is investigated as well. Furthermore, the encapsulation of an antitumor drug, 5-fluorouracil (5FU) within the hydrogels and its subsequent release in the presence and absence of H_2_O_2_ are also studied. Finally, cytotoxicity and growth inhibition of melanoma cancer cells B16F10 due to the release of 5FU from P[(EG_*n*_SA)_*x*_-*co*-HEA_*y*_] hydrogels under nonoxidative and oxidative conditions are evaluated.

## 2 Materials and Methods

### 2.1 Materials

Ethylene glycol diacrylate 90%, diethylene glycol diacrylate 75%, poly(ethylene glycol) diacrylate average *M*_n_ 250 as triethylene glycol diacrylate, 2,2′-thiodiethanethiol 90%, hydroxyethyl acrylate (HEA), Darocur 1173, and phosphate buffer solution (PBS) were purchased from Sigma-Aldrich and used as received. Dry dichloromethane 99.8% over molecular sieves, trimethylamine (NEt_3_), 1,8-diazabiciclo[5,4,0]undec-7-ene (DBU), and ethyl acetate were purchased from Fisher Scientific and used as received. Dulbecco's modified Eagle's medium (DMEM) supplemented with GlutaMAX, penicillin-streptomycin (5000 U/mL), and trypsin-EDTA (0.24%) phenol red was purchased from Gibco and used as received. Trypan blue solution, MTT (3-(4,5-dimethylthiazol-2-yl)-2,5-diphenyltetrazolium bromide), and dimethyl sulfoxide were purchased from Sigma-Aldrich, fetal bovine serum (FBS) from Life Technologies, and 5-fluorouracil from TCI.

### 2.2 Methods

#### 2.2.1 Synthesis of Acrylic thioether Monomers

The synthesis of the diacrylate thioether monomers was performed via a thiol-Michael addition click reaction with the following protocol. In an oven-dried round-bottom flask, 1 equiv of the desired poly(ethylene glycol) diacrylate was dissolved in dry dichloromethane using 50 mL of solvent for each 3.5 mmol of diacrylate starting materials. To this solution, 2 equiv of triethylamine and 0.05 equiv of DBU were added. To the resulting solution, 0.5 equiv of 2,2′-thiodiethanethiol was added dropwise under continuous stirring and static nitrogen atmosphere, keeping the temperature lower than 25 °C using an ice/water bath. After 4 h, the resulting mixture was put in ethyl acetate, using 250 mL for each 3.5 mmol of starting materials, extracted 3 times with water, using the same amount of ethyl acetate each time, and finally washed with the same amount of brine and, the organic part, dried over anhydrous sodium sulfate. The mixture was filtered, and the solvent was removed under vacuum to afford the pure products. Nuclear magnetic resonance (NMR) spectra were recorded at 25 °C temperature with a 300 MHz Bruker Avance III in CDCl_3_ (99.5% D) (Figures S1–S4). High-resolution mass spectrometry (HRMS) was measured with a Waters modelo SYNAPTTM G2 HDMSTM, using a Q-TOF detector and negative electrospray ionization ESI+, and elution of the sample was done using ACN:H_2_O 9:1 using 0.1% of formic acid (Figure S5).

#### 2.2.2 Hydrogel Formation

The PEG_*n*_SA (*n* = 1, 2 or 3) homopolymer hydrogels and P[(EG_*n*_SA)_*x*_-*co*-HEA_*y*_] (*n* = 1, 2 or 3) copolymer hydrogels with different percentages of HEA monomer (*y* = 80, 93 mol %) were formed in silicon molds of 6 mm diameter and 2 mm height by UV photopolymerization at 365 nm (80 mW/cm^2^). Previously, the monomers were mixed in a vial with 10 μL of Darocur 1173 used as the initiator. Then, the mixture was poured into the silicon mold and irradiated with UV light for 2 min for the homopolymers and 4 min for the copolymers.

#### 2.2.3 Swelling Tests

The P[(EG_*n*_SA)_*x*_-*co*-HEA_*y*_] hydrogels were swollen in 1 mL of PBS at pH 7.4 and room temperature for 24 h. Subsequently, the hydrogels were swollen under oxidative conditions by immersing them in 9 mM H_2_O_2_ for 4 h. Before starting the swelling tests, the hydrogels were weighted (*W*_0_). Then, the hydrogels were immersed in the swelling medium, and at established times, the samples were removed from the liquid, externally dried with filter paper to eliminate the excess liquid that could remain on the surface, and weighed (*W_t_*). The swelling percentage (*S*_w_) in wt % was calculated according to eq 1:
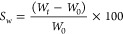
1

#### 2.2.4 Fourier Transform Infrared Spectroscopy

The hydrogels were swollen in PBS at pH 7.4 and room temperature for 24 h. Then, they were swollen in H_2_O_2_ 9 mM for 1, 2, and 4 h. FTIR spectra were recorded at each step using an FTIR spectrometer (Bruker INVENIO X).

#### 2.2.5 UV–Vis Spectrophotometry

Hydrogels were swollen in PBS at pH 7.4 for 24 h. In the case of the oxidized hydrogels, subsequently, they were swollen in H_2_O_2_ 9 mM for another 24 h. Then, they were placed between two quartz slides, and the absorbance was measured at 335 nm by using a Shimadzu UV-2550 spectrometer equipped with a film adapter.

#### 2.2.6 Rheological Measurements

Rheological measurements were carried out in an ARES-G2 rheometer (TA Instruments) at 37 °C. The P[(EG_3_SA)_*x*_-*co*-HEA_*y*_] hydrogels were swollen in PBS at pH 7.4 and room temperature for 24 h. In the case of the oxidized P[(EG_3_SA)_*x*_-*co*-HEA_*y*_] hydrogels, additionally they were swollen in H_2_O_2_ for 2 h before the measurement. Strain sweeps were performed from 0.01 to 100% strain at 1 Hz, and frequency sweeps were performed from 0.01 to 100 Hz at 1% strain.

#### 2.2.7 Digital Light 3D Printing (DLP)

Two different precursors were used for DLP. In the case of the pure EG_3_SA monomer, it was mixed with Darocur and poured into a cube basis of the DLP 3D printer (Asiga Max-UV, λ = 365 nm, 20 W/cm^2^), and 3D PEG_3_SA hydrogel structures were printed (layer height = 300 μm, exposure time = 30 s). For the copolymer, 20%mol EG_3_SA monomer was mixed with 80%mol HEA and Darocur and poured into the cube basis of the 3D printer leading to 3D P[(EG_3_SA)_20_-*co*-HEA_80_] hydrogel structures. The 3D-printed scaffolds were designed with Asiga Composer software.

#### 2.2.8 Drug Release Tests

P[(EG_3_SA)_*x*_-*co*-HEA_*y*_] hydrogels were washed with PBS for 7 days by replacing the washing solution daily to remove nonreacted monomers. First, 5-fluorouracil (5FU) was solved in PBS at pH 7.4 (1.5 mg/mL) by sonication for 7 min at 35 °C and encapsulated into the P[(EG_3_SA)_*x*_-*co*-HEA_*y*_] hydrogels by immersion for 24 h. After that, the supernatant was removed, and 5FU-loaded hydrogels were washed with PBS to remove the superficial drug and immersed into 1 mL of a fresh PBS solution with and without 9 mM H_2_O_2_ to start the drug delivery test. At specific times (1, 2, 4, 24, and 48 h), the supernatant was removed and replaced by 1 mL of a fresh PBS solution with and without 9 mM H_2_O_2_. The quantity of 5FU in the supernatant was determined by UV–vis Spectrophotometry (Shimadzu UV-2550 spectrometer) by recording the absorbance at 335 nm and comparing it with the 5FU calibration curve.

#### 2.2.9 *In Vitro* Cell Culture Tests

Prior to cell seeding, P[(EG_3_SA)_*x*_-*co*-HEA_*y*_] hydrogels were placed in a 48-well plate and sterilized under UV light for 30 min. Then, they were washed with 1 mL of PBS under sterile conditions for 7 days to remove nonreacted monomers by replacing the washing PBS solution daily. Subsequently, in the case of drug-loaded hydrogels, they were immersed into 1 mL of a 5FU solution (1.5 mg/mL in PBS pH 7.4) for 24 h under sterile conditions. After that, the supernatant was removed and the hydrogels were washed with 1 mL of PBS to remove the nonloaded drug. Then, nonloaded and 5FU-loaded hydrogels were incubated with 1 mL of fresh DMEM or the same media with H_2_O_2_ (1 or 0.1 mM) at 37 °C. At predetermined intervals, the supernatant was removed and replaced by 1 mL of fresh DMEM or the same media with H_2_O_2_ (1 or 0.1 mM).

Murine melanoma cells (B16F10) were cultured in Dulbecco’s modified Eagle’s medium (DMEM) enriched with 4500 mg/mL glucose and supplemented with 10% v/v fetal bovine serum (FBS), 2% v/v l-glutamine, 100 units/mL penicillin, and 100 mg/mL streptomycin on a 96 well-plate. B16F10 cells were seeded at a density of 5 × 10^4^ cells/mL on a 96-well plate and incubated at 37 °C (5% CO_2_ and 90% relative humidity) to confluence. After 24 h of incubation, the medium was replaced with the corresponding extracts and the mixtures incubated at 37 °C in humidified air with 5% CO_2_ for 24 h. A solution of MTS (0.5 mg/mL) was prepared in warm DMEM and added to the plate that was incubated at 37 °C for 4 h. Then, 0.1 mL of DMSO was added to each well and the absorbance was measured with a Cytation Bioteck using a test wavelength of 540 nm. The cell viability was calculated from eq 2:
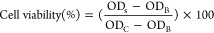
2where OD_S_, OD_B_, and OD_C_ are the optical density for the sample (S), blank (B), and control (C), respectively. Tests were performed in quadruplicate, and results are expressed as the mean ± standard deviation.

## 3 Results and Discussion

### 3.1 Synthesis and Characterization of Acrylic thioether Monomers (EG_*n*_SA)

**Figure 1:**
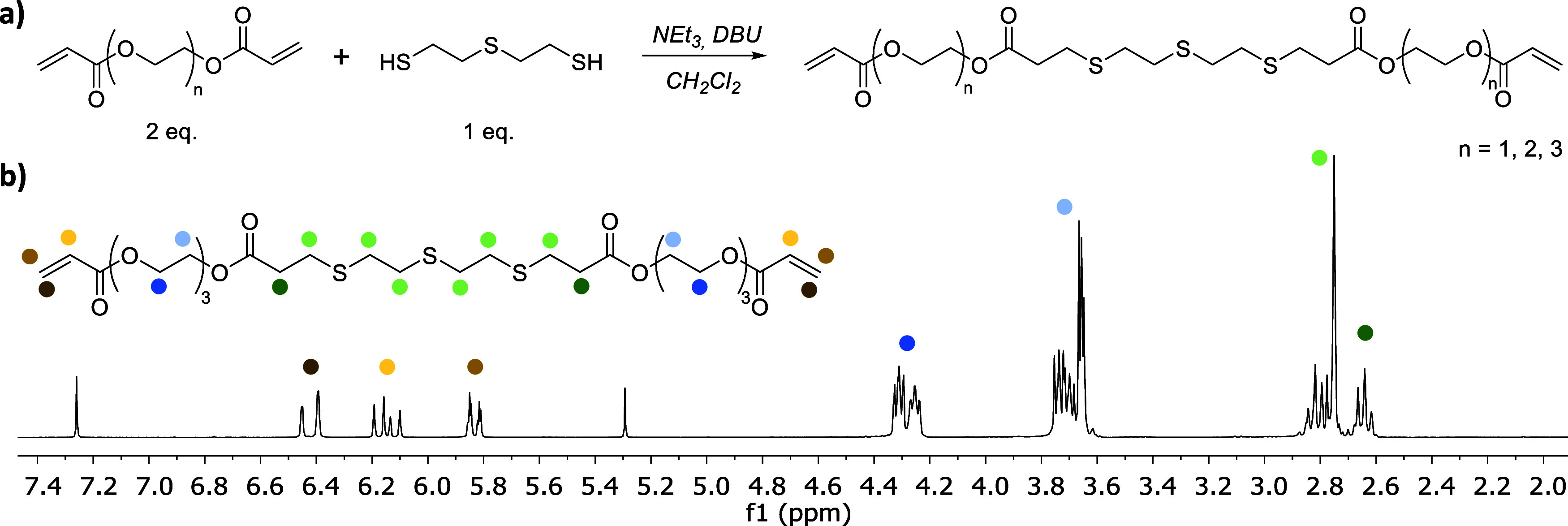
(a) Chemical route employed for the synthesis of acrylic thioether monomer EG_*n*_SA. (b) ^1^H NMR spectrum of the synthesized acrylic thioether monomer EG_3_SA.

The ethylene glycol sulfur diacrylate EG_*n*_SA monomers, with different lengths of the poly(ethylene glycol) EG_*n*_ segment (*n* = 1, 2, 3), were synthesized by thiol-Michael addition click reaction of 2 equiv of poly(ethylene glycol) diacrylate and 1 equiv of 2,2′-thiodiethanethiol using a NEt_3_ and DBU as catalyst (Figures 1a and S1–S3). The resulting monomers were characterized by ^1^H NMR (Figures 1b and S1–S3). The signals located in the region 2.6–2.9 ppm correspond to the protons associated with the thioether parts.^39^ The signals at 3.7 and 4.3 ppm are assigned to the ethylene oxide protons in the EG segment, whereas those in the region 5.8 to 6.4 ppm are attributed to the acrylate moieties.^40,41^ The coherent integration of the acrylate signals and the disappearance of the signals of the methylene group in alpfa to thiol confirm the success of the reaction.

**Figure 2:**
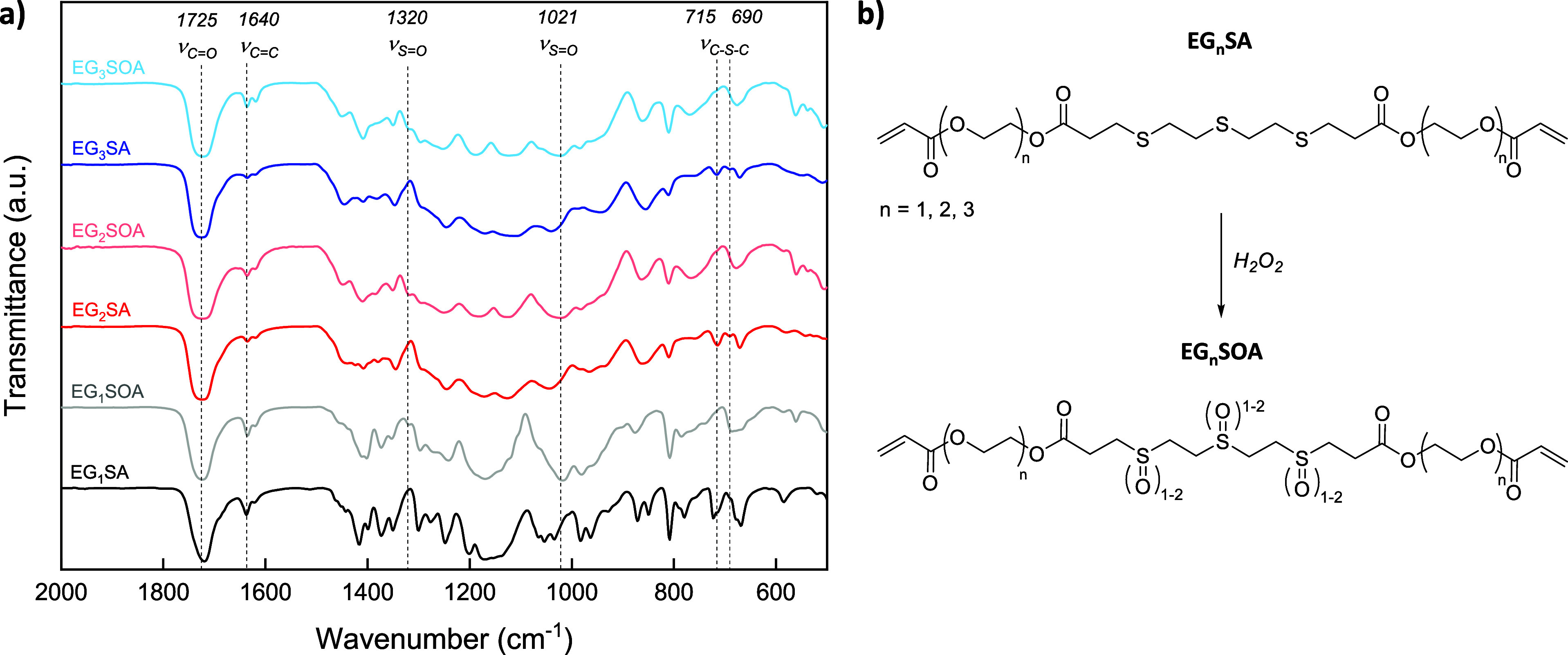
(a) FTIR spectra of the synthesized acrylic thioether monomers before (EG_*n*_SA) and after oxidation (EG_*n*_SOA) in the presence of H_2_O_2_, (*n* = 1, 2, 3). (b) Chemical route employed for the oxidation of the acrylic thioether monomers EG_*n*_SA (*n* = 1, 2, 3).

Then, the oxidation of the thioether-based monomers in the presence of oxidating agents (Figure 2), such as H_2_O_2_, into sulfoxide and/or sulfone groups (EG_*n*_SOA) was studied. The initial acrylic thioether monomers (EG_*n*_SA) were first characterized by FTIR spectroscopy (Figure 2a). The peaks at 1725 and 1640 cm^–1^ are attributed to C=O and C=C vibrations, respectively, confirming the presence of the acrylate groups into the molecular structure. Besides, the peaks at 690 and 715 cm^–1^ are the signatures of symmetric and asymmetric dimethyl sulfide bonds, respectively. After treatment with H_2_O_2_, FTIR spectra of the oxidized monomers (EG_*n*_SOA) exhibited an additional peak at 1021 cm^–1^ corresponding to the stretching of the double bond S=O in sulfoxides, together with another peak at 1320 cm^–1^ that can be assigned to S=O in sulfones (Figure 2a). In addition to this, the signals of the sulfide peaks (C–S–C) disappeared and the signal of the acrylate groups was retained which indicates the successful oxidation of the thioether.^42,43^ These results were corroborated by ^1^H NMR spectroscopy (Figure S6). The oxidation of the EG_3_SA monomer in the presence of H_2_O_2_ for 4 h gave rise to the appearance of a new band at 3.0–3.4 ppm that is ascribed to alpha-protons of sulfoxides and sulfones.^25,44^

**Figure 3:**
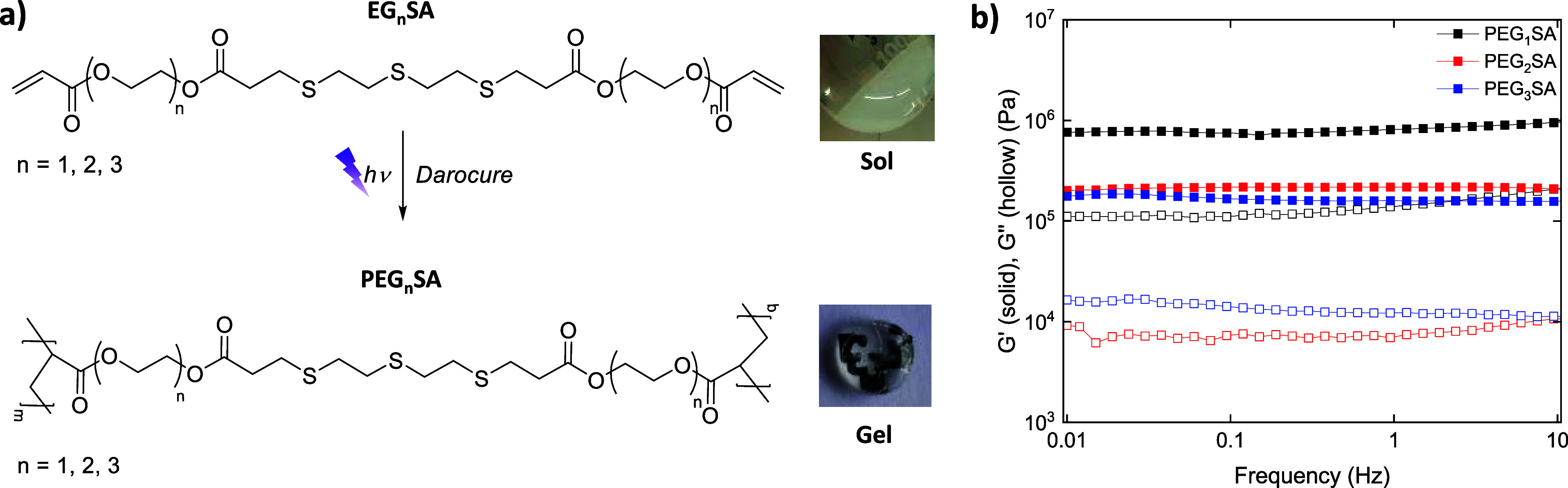
(a) Hydrogel formation by photopolymerization of EG_*n*_SA (*n* = 1, 2, 3) with pictures of the sol precursor and the photopolymerized gel. (b) Evolution of the elastic modulus (*G*′) and loss modulus (*G*′′) of PEG_*n*_SA hydrogels as a function of the frequency.

The diacrylate thioether monomers were photopolymerized by UV light using Darocure as a photoinitiator, leading to the formation of hydrogels (Figure 3a). The hydrogel formation was determined by dynamic oscillatory rheological measurements. Rheological properties of the hydrogel were characterized as a function of the length of the EG chains. First, the linear viscoelastic regime (LVR) in the hydrogels was determined by strain sweeps (Figure S7). At low strains, the elastic modulus (*G*′) was higher than the loss modulus (*G*″), which is the condition for the gel formation. However, at high strains, this behavior was reversed, and the samples passed from a solid-like to a liquid-like state. It was observed that the deformation at break (γ_0_) depended on the length of the EG chain. PEG_1_SA hydrogels exhibited a γ_0_ ≈ 5% strain, which increased up to γ_0_ ≈25% strain for PEG_3_SA hydrogels due to the enhanced flexibility of the hydrogels with the length of the EG segment. Then, the frequency sweeps in the LVR showed that *G*′ was higher than *G*″ in all the frequency ranges and independent of the frequency (Figure 3b). Besides, a decrease of the elastic modulus was also observed with the length of the EG chain from ≈8.1 × 10^5^ Pa up to ≈1.6 × 10^5^ Pa for PEG_1_SA and PEG_3_SA hydrogels, respectively. A key feature of these hydrogels is the ability of the thioether groups to be oxidized in the presence of ROS triggers, such as H_2_O_2_, into sulfoxide and/or sulfone groups with a higher water absorption capability during swelling. Although the PEG_*n*_SOA hydrogels experienced a higher swelling than nonoxidized PEG_*n*_SA hydrogels, they were brittle and their network structure was totally disintegrated after 1 h of oxidation (Figure S8), which limits their functional applications.

### 3.2 Synthesis and Characterization of Hydrogels Based on Acrylic thioether Copolymers P[(EG_*n*_SA)-*co*-HEA]

**Figure 4:**
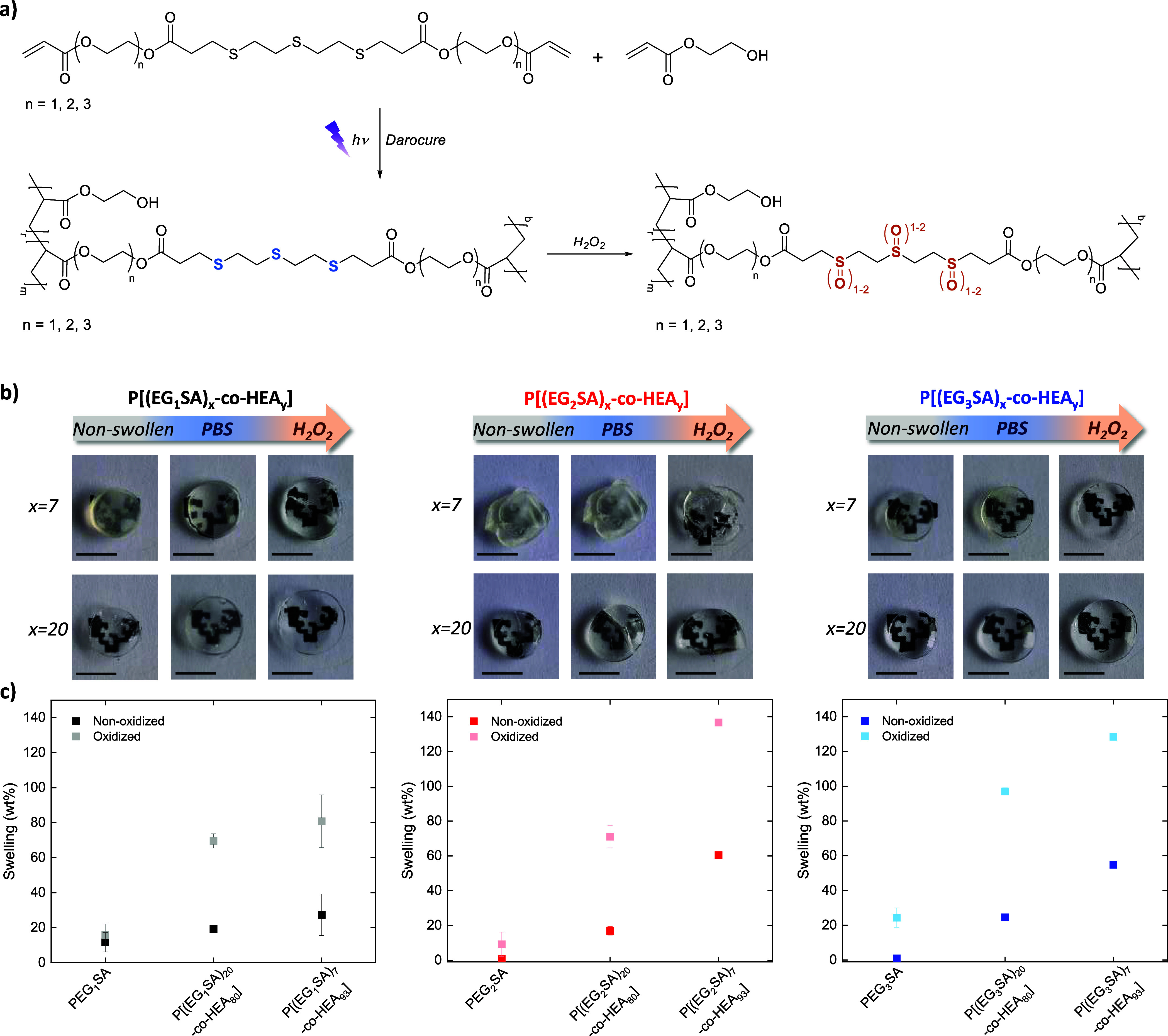
(a) Hydrogels formation by photopolymerization of EG_*n*_SA (*n* = 1, 2, 3) in the presence of 2-hydroxyethyl acrylate (HEA), and the chemical route employed for the oxidation and swelling of the P[(EG_*n*_SA)_*x*_-*co*-HEA_*y*_] hydrogels induced by H_2_O_2_, (*x* = 7, 20). (b) Pictures of the swollen P[(EG_*n*_SA)_*x*_-*co*-HEA_*y*_] hydrogels in PBS (pH 7.4) under nonoxidative conditions and in the presence of 9 mM H_2_O_2_. Scale bars = 5 mm. (c) Swelling evolution of the nonoxidized and oxidized P[(EG_*n*_SA)_*x*_-*co*-HEA_*y*_] hydrogels.

To overcome the abovementioned mechanical limitations, the synthesized acrylic thioether monomers EG_*n*_SA (*n* = 1, 2, 3) were copolymerized with different polar monomers, including 2-hydroxyethyl acrylate (HEA), and [2-(acryloyloxy)ethyl]trimethylammonium chloride (AETAC), and polymers such as poly(ethylene glycol diacrylate) (PEGDA) and poly(ethylene glycol methacrylate) (PEGMA). The monofunctional acrylic monomers and the acrylic polymers can act as internal diluents and flexibilizers of the hydrogel network (Figures 4a and S9). Due to their polar nature, the acrylic comonomers helped increase the polarity of the hydrogels and therefore improve the compatibility in aqueous media. In all cases, hydrogels were successfully formed. The gel fraction of all hydrogels is 100 wt % because both the monomer EG_*n*_SA and comonomers employed are in the liquid state and are miscible between them without the addition of any solvent. Then, the ROS response of the copolymer networks was evaluated. The hydrogels copolymerized with PEGMA and AETAC were totally disintegrated during swelling in the presence of H_2_O_2_, and those copolymerized with PEDGA were brittle. Interestingly, the hydrogels copolymerized with HEA, P[(EG_*n*_SA)_*x*_-*co*-HEA_*y*_], presented a totally different behavior without breaking during the oxidative swelling (9 mM H_2_O_2_ for 4 h). This can be attributed to the fact that AETAC, with charged ammonium groups, and PEGMA and HEA with hydroxyl end-groups are more polar than PEDGA. Besides, the charged ammonium groups of AETAC gave it the highest polar properties, allowing P[(EG_2_SA)-*co*-AETAC] hydrogels to hold more water, leading to a water pressure-induced break. In the case of P[(EG_2_SA)-*co*-PEGMA] and P[(EG_2_SA)-*co*-HEA] hydrogels with hydroxyl end groups, the longer chains of PEGMA (*M*_n_ = 360 Da) gave rise to less cross-linked hydrogels, P[(EG_2_SA)-*co*-PEGMA], than those prepared with HEA (*M*_w_ = 116.12 Da), thus allowing them to hold more water and making also more brittle than P[(EG_2_SA)-*co*-HEA] hydrogels. The influence of the HEA concentration on the swelling behavior of the P[(EG_*n*_SA)_*x*_-*co*-HEA_*y*_] hydrogels under nonoxidative and oxidative conditions was further studied in more detail (Figure 4b,c). The swelling of the PEG_*n*_SA hydrogels mimicking normal physiological conditions, in phosphate buffer solution (PBS) at pH 7.4, was very low (≤10 wt %), but increased with the percentage of HEA in the copolymer reaching values of 30 wt % for P[(EG_1_SA)_7_-*co*-HEA_93_] and 60 wt % for P[(EG_2_SA)_7_-*co*-HEA_93_] and P[(EG_3_SA)_7_-*co*-HEA_93_] due to the higher flexibility of the hydrogel network. Under oxidative conditions, in the presence of H_2_O_2_, PEG_*n*_SA hydrogels were fully disintegrated, whereas P[(EG_*n*_SA)_*x*_-*co*-HEA_*y*_] hydrogels presented a considerably higher swelling ability, which was even more pronounced in the case of hydrogels synthesized with the acrylic thioether monomer (EG_*n*_SA) with longer EG segments (*n* = 2, 3). Thus, the swelling ability increased up to ≈80 wt % for P[(EG_1_SA)_7_-*co*-HEA_93_] hydrogels in the presence of H_2_O_2_, ≈140 wt % for P[(EG_2_SA)_7_-*co*-HEA_93_], and 130 wt % for P[(EG_3_SA)_7_-*co*-HEA_93_] hydrogels. However, P[(EG_2_SA)_*x*_-*co*-HEA_*y*_] hydrogels did not remain stable during the oxidative swelling and were partially broken, being discarded. Therefore, P[(EG_3_SA)_*x*_-*co*-HEA_*y*_] hydrogels were selected as ROS-responsive matrices for further drug release experiments. The oxidation of these thioether-based polymer hydrogels into sulfoxide or sulfone groups in the presence of H_2_O_2_ was assessed by FTIR measurements (Figure S10). FTIR spectra of the oxidized polymer hydrogels exhibited a peak at 1320 cm^–1^ that can be assigned to the formation of sulfones of O=S=O in the case of both the homopolymer and copolymers, together with the peak at 1041 cm^–1^ corresponding to the stretching of the double bond S=O in sulfoxides in the case of the oxidized homopolymer PEG_3_SOA.

**Figure 5:**
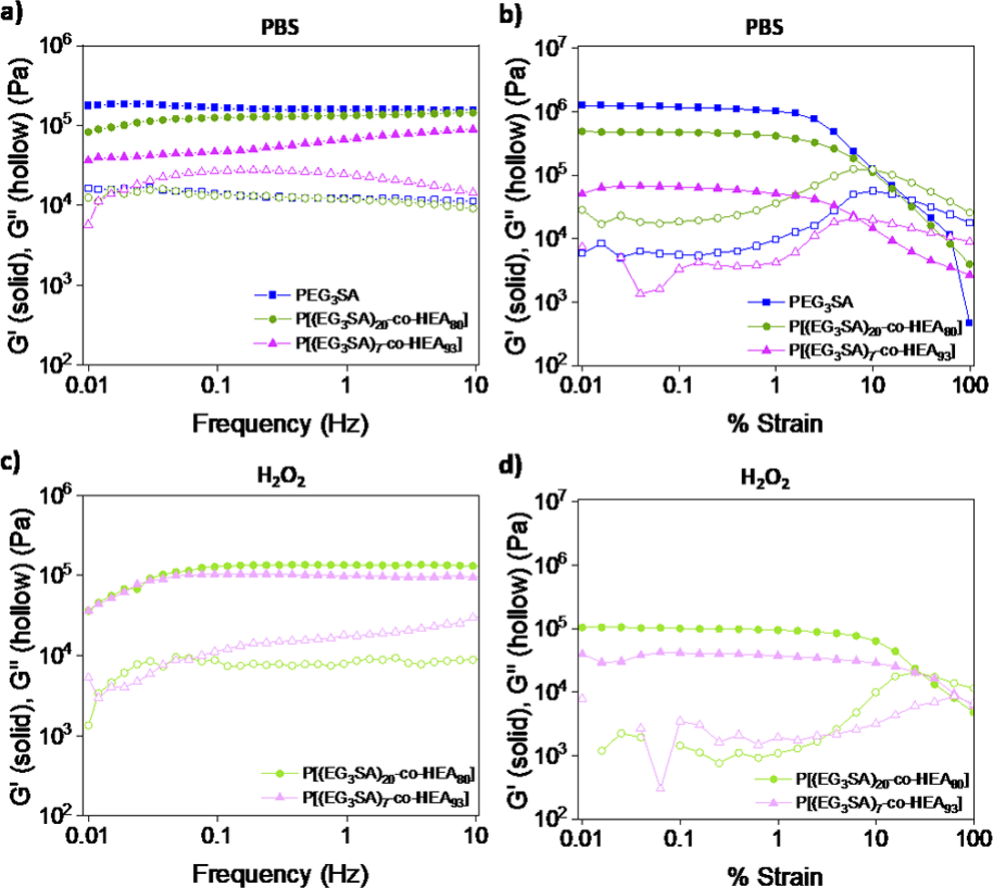
(a) Frequency sweeps at 1% strain and (b) strain sweeps at 1 Hz of PEG_3_SA, P[(EG_3_SA)_20_-*co*-HEA_80_], and P[(EG_3_SA)_7_-*co*-HEA_93_] hydrogels swollen in PBS under nonoxidative conditions. (c) Frequency sweeps at 1% strain and (d) strain sweeps at 1 Hz of swollen P[(EG_3_SA)_20_-*co*-HEA_80_] and P[(EG_3_SA)_7_-*co*-HEA_93_] hydrogels in the presence of 9 mM H_2_O_2_.

The effects of the incorporation of HEA on the mechanical properties of the P[(EG_3_SA)_*x*_-*co*-HEA_*y*_] hydrogels were evaluated by dynamic oscillatory rheological measurements (Figure 5). In all cases, under nonoxidative conditions (in PBS), *G*′ was higher than *G*″ for all frequency range (Figure 5a). The elastic modulus decreased with the HEA percentage from *G*′ ≈ 1.3 × 10^5^ Pa for P[(EG_3_SA)_20_-*co*-HEA_80_] to *G*′ ≈ 6.7 × 10^4^ Pa for P[(EG_3_SA)_7_-*co*-HEA_93_], while the elongation at break (γ_0_) slightly decreased from 10% strain to 7% strain (Figure 5b). Under oxidative conditions (in H_2_O_2_), when the hydrogels have achieved the maximum swelling capacity, PEG_3_SA hydrogels that did not contain HEA were totally disintegrated, and their mechanical properties could not be determined. In the case of P[(EG_3_SA)_*x*_-*co*-HEA_*y*_] hydrogels, both samples exhibited the same behavior with *G*′ higher than *G*′′ in all the frequency ranges without observing significant differences in the values of the elastic modulus (*G*′ ≈ 10^5^ Pa) between them (Figure 5c). Nevertheless, the elongation at break (γ_0_) highly improved reaching values of 25% strain for P[(EG_3_SA)_20_-*co*-HEA_80_] and 65% strain for P[(EG_3_SA)_7_-*co*-HEA_93_] (Figure 5d) due to the combination of two factors, on the one hand, the flexibility increase of the hydrogel network due to the presence of HEA, and on the other hand the more hydrophilic character of the sulfoxide and sulfone groups formed during the EG_3_SA oxidation (Figure 2b). These values of elastic modulus are in the range of those of the dermis and subcutaneous skin, making them attractive candidates for potential applications as dermal patches.^45−47^

The degradation of P[(EG_3_SA)_*x*_-*co*-HEA_*y*_] hydrogels over time was also studied (Figure S11). Under nonoxidative conditions, P[(EG_3_SA)_7_-*co*-HEA_93_] hydrogels only swelled over time, reaching a plateau after 14 days, but they remained stable for 21 days at least. P[(EG_3_SA)_20_-*co*-HEA_80_] hydrogels did show any significant degradation over 7 days, when they started to disintegrate losing 25% of their initial weight after 21 days, which can be attributed to the high capacity of the hydrogels to hold water due to their polarity, while they are less flexible because they are more cross-linked, leading to water pressure-induced disintegration. Under oxidative (H_2_O_2_) conditions, the degradation of P[(EG_3_SOA)_7_-*co*-HEA_93_] hydrogels was very low experiencing only 10 wt % weight loss after 21 days due to the low percentage of ROS-responsive EG_3_SA monomer within the copolymer. Otherwise, although P[(EG_3_SOA)_20_-*co*-HEA_80_] hydrogels exhibited only 10 wt % weight loss over 7 days, they later started to suffer a more significant degradation, losing up to 30 wt % of their initial weight after 21 days. The less flexible nature of this network due to its higher reticulation together with its higher ROS-response ability allowed it to hold more water, leading to a faster disintegration.

**Figure 6:**
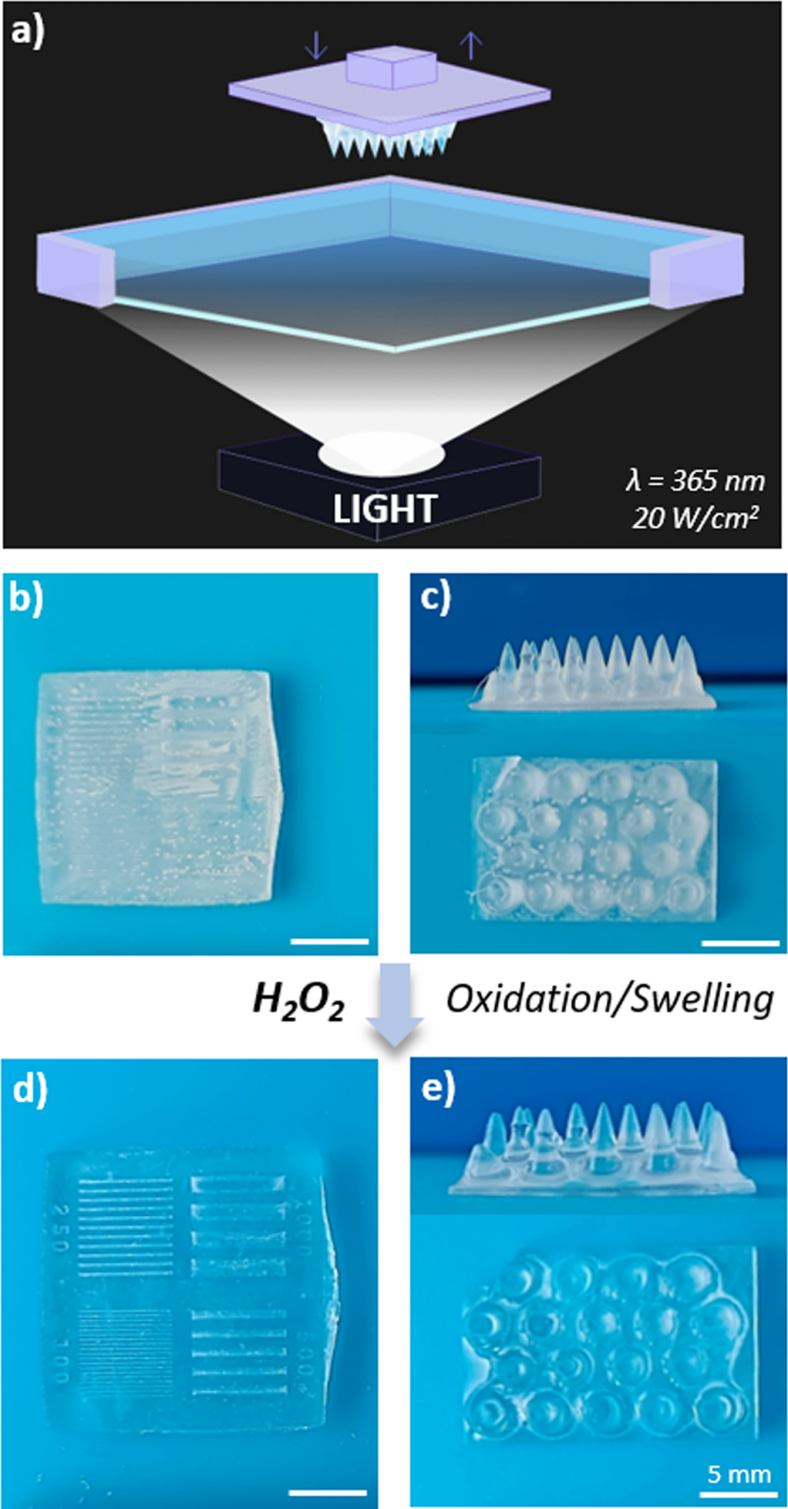
(a) Schematic representation of the DLP process employed to print the hydrogels. Shape-defined 4D-printed P[(EG_3_SA)_20_-*co*-HEA_80_] hydrogel scaffolds: (b) lined holes of variable line widths and (c) needles. Shape-defined 4D-printed P[(EG_3_SOA)_20_-*co*-HEA_80_] hydrogel scaffolds after swelling in the presence of 9 mM H_2_O_2_: (d) lined holes of variable line widths and (e) needles. Scale bars = 5 mm.

The design of materials with the ability to be processed into 3D scaffolds with complex structures is actively searched in the biomedical field for tissue engineering purposes.^48−50^ In this regard, P[(EG_3_SA)_*x*_-*co*-HEA_*y*_] hydrogels not only presented an enhanced elastic behavior but also could be successfully processed through digital light printing (DLP) (Figure 6a). First, the DLP parameters were optimized by printing 3D square scaffolds (15 mm × 15 mm × 2 mm) with four different sets of lined holes of variable line widths (100, 250, 500, and 1000 μm). The printing resolution increased with the percentage of thioether acrylate (EG_3_SA) in the copolymer (Figures 6b and S12). Higher resolution printed lined holes were obtained in the case of P[(EG_3_SA)_20_-*co*-HEA_80_] gels than for P[(EG_3_SA)_7_-*co*-HEA_93_] gels. Then, the thioether acrylic monomers were also used to print more complex morphologies like needles (3.5 mm height) over a square base (15 × 10 mm), pointing out a higher printing integrity of P[(EG_3_SA)_20_-*co*-HEA_80_] than P[(EG_3_SA)_7_-*co*-HEA_93_] gels (Figures 6c and S13). In addition, the printed hydrogels possessed stimuli-responsive properties due to the presence of thioether groups in the polymer chain, which modulated the oxidation and swelling behavior in the presence of ROS, making them 4D-printable hydrogels. Thus, 4D-printed PEG_3_SA hydrogels were totally disintegrated after swelling under oxidative conditions (Figure S13a), and the 4D-printed scaffolds made with the copolymer P[(EG_3_SA)_*x*_-*co*-HEA_*y*_] were capable of swelling in the three dimensions (≈120 wt % swelling) retaining their morphology with high-fidelity and exhibiting a high-transparency (Figure 6d,e and Figure S13b).

### 3.3 *In Vitro* Drug Release Experiments

**Figure 7:**
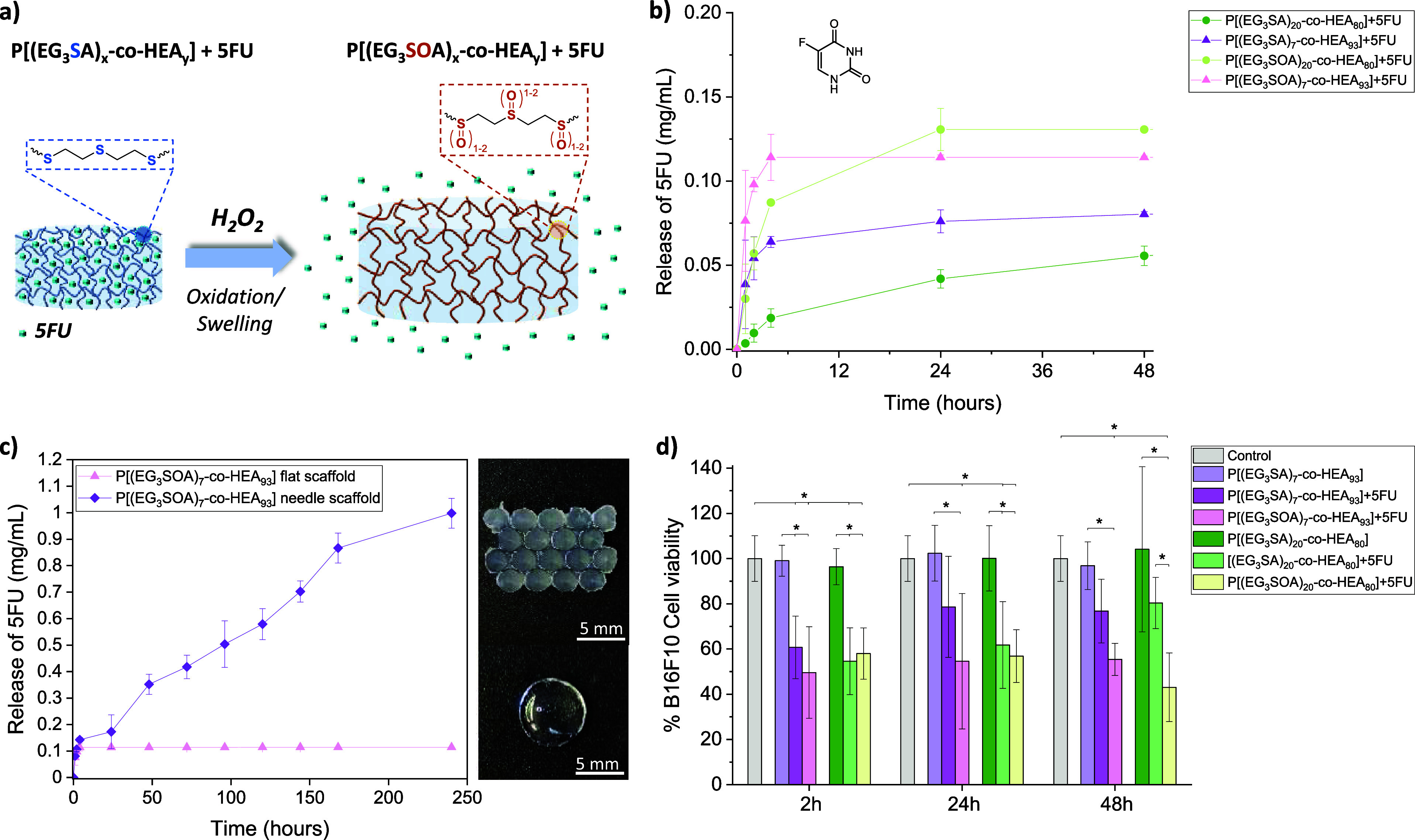
(a) Schematic representation of the 5FU release from P[(EG_*n*_SA)_*x*_-*co*-HEA_*y*_] hydrogels induced by oxidation and swelling in the presence of H_2_O_2_. (b) Release of 5FU from hydrogels under nonoxidative conditions in PBS, P[(EG_3_SA)_20_-*co*-HEA_80_] and P[(EG_3_SA)_7_-*co*-HEA_93_], and oxidative conditions in the presence of 9 mM H_2_O_2_, P[(EG_3_SOA)_20_-*co*-HEA_80_] and P[(EG_3_SOA)_7_-*co*-HEA_93_]. (c) Release of 5FU from printed P[(EG_3_SOA)_7_-*co*-HEA_93_] hydrogels with different geometries, flat surface scaffold and needle scaffold, under oxidative conditions in the presence of 9 mM H_2_O_2_, including representative pictures of these scaffolds. (d) *In vitro* cytotoxicity tests of 5FU release from P[(EG_3_SA)_20_-*co*-HEA_80_], and P[(EG_3_SA)_7_-*co*-HEA_93_] hydrogels in contact with B16F10 cells under nonoxidative conditions in PBS, and oxidative conditions in the presence of 1 mM H_2_O_2_. Diagrams include the mean and standard deviation (*n* = 3) and the ANOVA results at a significance level of *p < 0.5 using the Tukey’s test.

P[(EG_3_SA)_*x*_-*co*-HEA_*y*_] hydrogels were further explored as ROS-responsive matrices for the encapsulation of an anticancer drug, 5-fluorouracil (5FU), which is used for the treatment of skin cancer, among other cancer types.^51,52^ First, the delivery of 5FU was studied by mimicking normal physiological conditions (in PBS, pH 7.4) (Figure 7a,b). P[(EG_3_SA)_7_-*co*-HEA_93_] hydrogels presented the highest release of 5FU in the first 2 h reaching a plateau after 24 h, whereas P[(EG_3_SA)_20_-*co*-HEA_80_] hydrogels, which were more reticulated due to the higher percentage of diacrylate sulfur monomer, presented the highest release of 5FU in 24 h reaching a plateau. In the presence of ROS, such as H_2_O_2_, the release rate of 5FU was faster due to the higher swelling of the hydrogels under oxidative conditions (Figure 4c). P[(EG_3_SA)_7_-*co*-HEA_93_] hydrogels, which possessed a higher swelling ability under oxidative conditions (≈130 wt %) than P[(EG_3_SA)_20_-*co*-HEA_80_] hydrogels (≈100 wt %) (Figure 4c), also presented a higher release rate of 5FU in the first hours, reaching a plateau (≈0.12 mg/mL) after 4 h and a 2-fold increase in comparison with the release in PBS (≈0.06 mg/mL). Besides, the influence of the geometry/size of the printed P[(EG_3_SA)_7_-*co*-HEA_93_] hydrogels on the drug delivery properties was also studied (Figure 7c). In the case of flat surface cylinders of 55 mm^2^ surface area and 20 mm^3^ volume, the release of 5FU (≈0.12 mg/mL) took place in the first 4 h. By increasing the surface area up to 500 mm^2^ and the volume up to 277 mm^3^ through the printing of cone-type needles, we were able to increase the load of 5FU and achieved a more sustained and a 9-fold increase in the drug release over time. The cytotoxicity effect of 5FU release from P[(EG_3_SA)_*x*_-*co*-HEA_*y*_] hydrogels was later assessed *in vitro* with murine melanoma cells (B16F10) (Figure 7d). Nonloaded hydrogels did not induce any decrease in the B16F10 cell viability in comparison with cells only treated with culture media used as control, which proved that they are noncytotoxic. On the other hand, the drug released from 5FU-loaded P[(EG_3_SA)_*x*_-*co*-HEA_*y*_] hydrogels under normal physiological conditions (PBS) gave rise to a decrease in the B16F10 cell viability. In the case of 5FU-loaded P[(EG_3_SA)_7_-*co*-HEA_93_] hydrogels, the B16F10 cell viability decreased up to ≈60% for 2 h and ≈80% after 48 h. For 5FU-loaded P[(EG_3_SA)_20_-*co*-HEA_80_] hydrogels, it decreased up to ≈55% for 2 h, ≈60% for 24 h, and ≈80% after 48 h. Interestingly, the death of B16F10 cancer cells was enhanced in the presence of H_2_O_2_, a kind of ROS that is overproduced in cancer areas.^20,21^ In the case of P[(EG_3_SA)_7_-*co*-HEA_93_] hydrogels, the B16F10 cell death was modulated by the 5FU release profile over time, leading to a ≈50% decrease in the B16F10 cell viability after 2 h and ≈55% decrease after 48 h. Concerning P[(EG_3_SA)_20_-*co*-HEA_80_] hydrogels that presented an enhanced swelling behavior in the presence of ROS, the cell viability decreased up to ≈55% for 24 h and ≈40% after 48 h. Overall, the P[(EG_3_SA)_*x*_-*co*-HEA_*y*_] hydrogels can act as ROS scavenger agents, as well as their tunable mechanical and swelling properties allowed to modulate the release of 5FU and the B16F10 cell viability over time, which opens the route to the development of dermal patches for topical treatment of cancer. Table 1 summarizes the resulting mechanical and biological properties of the P[(EG_3_SA)_*x*_-*co*-HEA_*y*_] hydrogels.

**Table 1:** Summary of the Mechanical and Biological Properties of the Hydrogels P(EG_3_SA) and P[(EG_3_SA)_*x*_-co-HEA_*y*_] under nonoxidative (PBS) and Oxidative (H_2_O_2_) Conditions

hydrogel	swelling (wt %)		*G*′ (Pa)		yield strain (%)		drug release (mg/mL)		% B16F10 cell viability		
	*PBS*	H_2_O_2_	PBS	H_2_O_2_	PBS	H_2_O_2_	PBS	H_2_O_2_	PBS	5FU-PBS	5FU-H_2_O_2_
P(EG_3_SA)	2	27	1.4 × 10^5^								
P[(EG_3_SA)_20_-*co*-HEA_80_]	23	100	1.3 × 10^5^	1 × 10^5^	10	25	0.06	0.14	110	80	50
P[(EG_3_SA)_7_-*co*-HEA_93_]	60	130	6.7 × 10^5^	1 × 10^5^	7	65	0.07	0.12	98	80	62

## 4 Conclusions

Aqueous soluble ethylene glycol sulfur diacrylate EG_*n*_SA monomers, with different lengths of the poly(ethylene glycol) EG_*n*_ segment (*n* = 1, 2, 3), were successfully synthesized by thiol-Michael addition click reaction. Their UV-light induced photopolymerization produced PEG_*n*_SA hydrogels whose flexibility could be modulated by the length of the EG_*n*_ segment, which increased from PEG_1_SA (*G*′ ≈ 8.1 × 10^5^ Pa) to PEG_3_SA (*G*′ ≈ 1.6 × 10^5^ Pa) hydrogels because of the decrease of the elastic modulus as determined by rheology. The mechanical stability of the hydrogels under oxidative swelling conditions was enhanced by the copolymerization of EG_*n*_SA monomers with a polar comonomer, 2-hydroxyethyl acrylate (HEA), leading to P[(EG_*n*_SA)_*x*_-*co*-HEA_*y*_] (*x* = 3, 20) hydrogels with higher compatibility in aqueous media and elasticity (*G*′ ≈ 6.7 × 10^4^ Pa for P[(EG_3_SA)_7_-*co*-HEA_93_]), making them less brittle materials.

Interestingly, the thioether hydrogels exhibited a superior swelling ability in the presence of ROS triggers than under nonoxidative conditions. Thus, the swelling of the hydrogels increased in the presence of ROS (i.e., H_2_O_2_), achieving a ≈130 wt % swelling for P[(EG_3_SA)_7_-*co*-HEA_93_]. In addition to this, the combined ability of these EG_*n*_SA monomers to be photopolymerized by UV light together with their ROS response allowed their processing through advanced 4D printing techniques giving rise to ROS-responsive shape-defined hydrogels.

The P[(EG_3_SA)_*x*_-*co*-HEA_*y*_] hydrogels were employed as matrixes for the encapsulation of an antitumor drug, 5-fluorouracil (5FU), whose release induced the decrease in cell viability of melanoma cancer cells B16F10, in a range of 40–60% that was modulated by the EG_3_SA percentage and the presence or absence of ROS. Overall, these results prove the excellent ROS-responsive properties of the acrylic thioether-based hydrogels for smart drug release for the potential treatment of localized pathologies.

## Supporting Information Available

^1^H NMR and ^13^C NMR spectra of EG_*n*_SA monomers in CDCl_3_, UPLC-MS spectrum and chromatogram of EG_3_SA monomer, rheological strain sweeps of PEG_*n*_SA hydrogels, pictures and swelling tests of PEG_*n*_SA hydrogels, photopolymerization and oxidation tests of EG_2_SA with different monomers, FTIR spectra of PEG_3_SA and P[(EG_3_SA)_7_-*co*-HEA_93_] hydrogels before and after oxidation, degradation assay of PEG_3_SA and P[(EG_3_SA)_7_-*co*-HEA_93_] hydrogels, and 4D printing tests of PEG_3_SA and P[(EG_3_SA)_7_-*co*-HEA_93_] hydrogels (PDF).
