# Supplementary material for: ROS-Responsive 4D Printable Acrylic Thioether-Based Hydrogels for Smart Drug Release
Source: Chem Mater. 2023 Dec 13;36(3):1262–72. doi: 10.1021/acs.chemmater.3c02264 (PMC10870821; doi:10.1021/acs.chemmater.3c02264)
Supplement: Supplementary file 1 — cm3c02264_si_001.pdf [file cm3c02264_si_001.pdf]

SUPPORTING INFORMATION

ROS-responsive 4D printable acrylic thioether-based hydrogels for smart drug release

Maria Regato-Herbella,[1,2] Isabel Morhenn,[1] Daniele Mantione,[1,3] Giuseppe Pascuzzi,[4] Antonela Gallastegui,[1] Beatriz Valle Caribe Dos Santos,[2] Sergio E. Moya,[2] Miryam Criado-Gonzalez,[1] David Mecerreyes[1,3*]*

[1] POLYMAT University of the Basque Country UPV/EHU, Joxe Mari Korta Center. Avda. Tolosa 72, 20018, Donostia-San Sebastián, Spain

[2] Center for Cooperative Research in Biomaterials (CIC biomaGUNE), Basque Research and Technology Alliance (BRTA). Paseo de Miramón 194, 20014, Donostia-San Sebastián, Spain.

[3] Ikerbasque, Basque Foundation for Science. 48013 Bilbao, Spain

[4] Department of Chemistry, Materials and Chemical Engineering “Giulio Natta” Politecnico di Milano, Piazza Leonardo da Vinci 32, 20133 Milano (Italy)

Synthesis of 4,16-dioxo-3,17-dioxa-7,10,13-trithianonadecane-1,19-diyl diacrylate (EG_1SA)

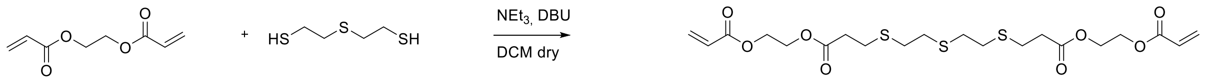

^{1}H NMR (300 MHz, CDCl3) δ 6.54 – 6.35 (m, 2H), 6.14 (dd, J = 17.3, 10.4 Hz, 2H), 5.86 (ddd, J = 10.4, 2.4, 1.5 Hz, 2H), 4.46 – 4.26 (m, 8H), 2.90 – 2.71 (m, 12H), 2.69 – 2.61 (m, 4H). FT-IR ν_{max}/cm^{-1} 3003 (=C-H), 2948, 2939 (C-H), 1718 (C=O) and 1415 (C=C), 1170, 809.

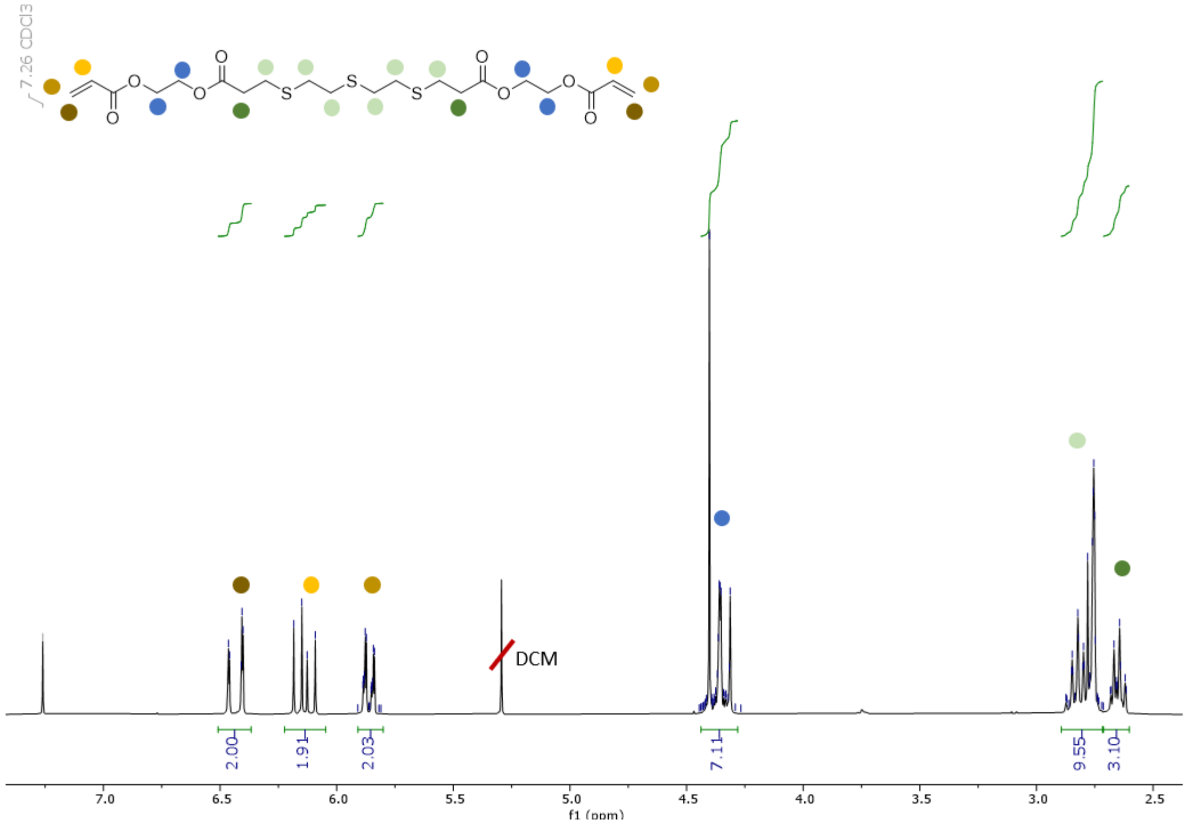

Figure S1. ^{1}H NMR spectrum of EG_1SA in $CDCl_3$.

Synthesis of 7,19-dioxo-3,6,20,23-tetraoxa-10,13,16-trithiapentacosane-1,25-diyl diacrylate (EG2SA)

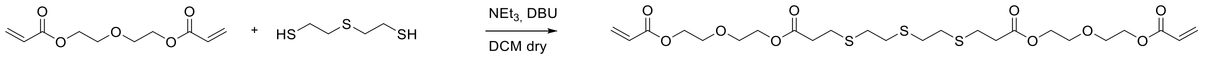

^{1}H NMR (300 MHz, CDCl3) δ 6.43 (dt, J = 17.3, 1.7 Hz, 2H), 6.15 (ddd, J = 17.3, 10.4, 2.0 Hz, 2H), 5.84 (ddd, J = 10.4, 3.3, 1.5 Hz, 2H), 4.46 – 4.11 (m, 8H), 3.89 – 3.51 (m, 8H), 2.91 – 2.72 (m, 12H), 2.72 – 2.49 (m, 4H). FT-IR ν_{max}/cm^{-1} 3002 (=C-H), 2955, 2917 (C-H), 1726 (C=O) and 1402 (C=C), 1171, 809.

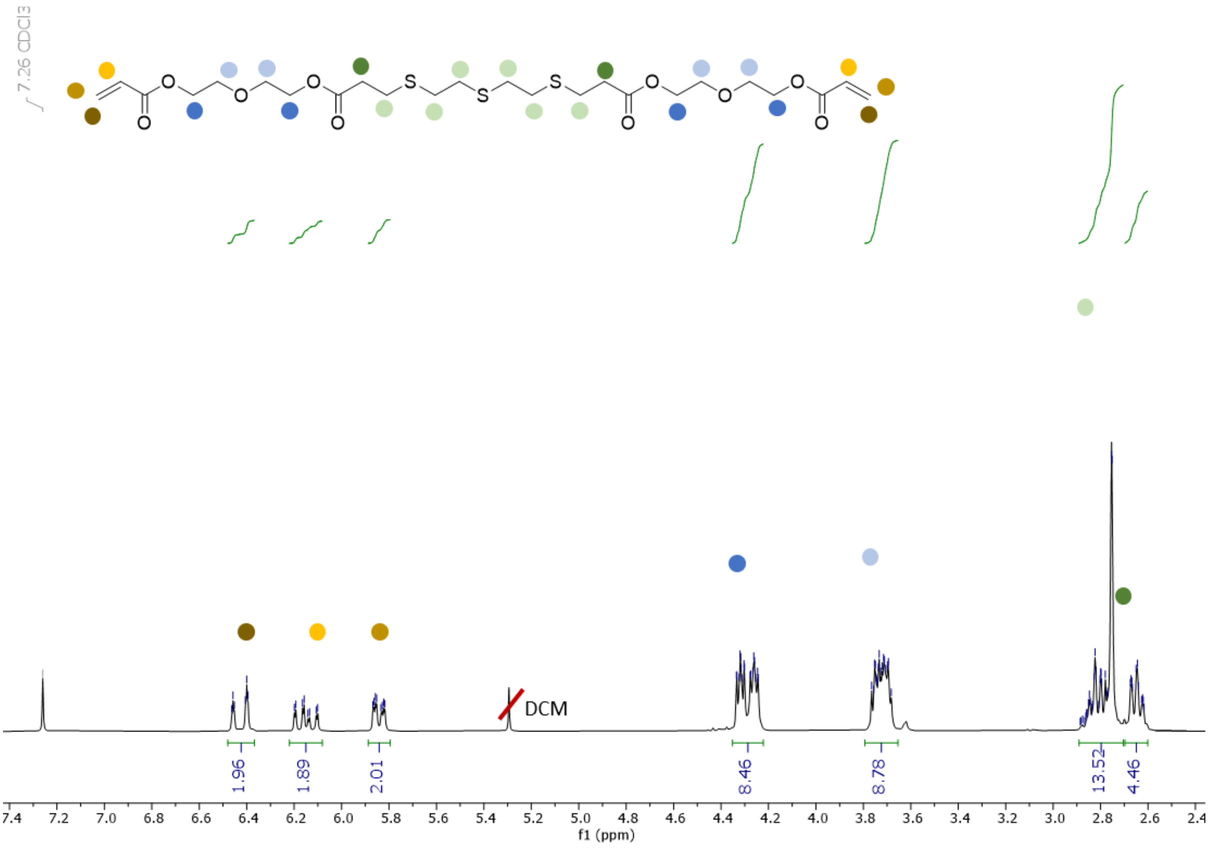

Figure S2. ^{1}H NMR spectrum of EG_2SA in $CDCl_3$.

Synthesis of 10,22-dioxo-3,6,9,23,26,29-hexaoxa-13,16,19-trithiahentriacontane-1,31-diyl diacrylate (EG3SA)

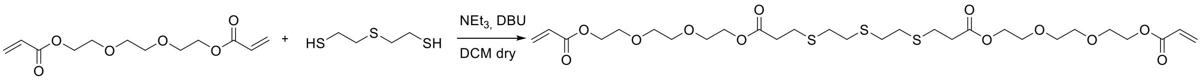

^{1}H NMR (300 MHz, CDCl3) δ 6.48 – 6.36 (m, 2H), 6.14 (ddd, J = 17.3, 10.4, 0.9 Hz, 2H), 5.83 (dt, J = 10.4, 1.8 Hz, 2H), 4.28 (dtd, J = 17.6, 4.6, 1.3 Hz, 8H), 3.80 – 3.56 (m, 16H), 2.88 – 2.69 (m, 12H), 2.64 (t, J = 7.2 Hz, 4H). FT-IR ν_{max}/cm^{-1} 3010 (=C-H), 2953, 2910, 2864, (C-H), 1726 (C=O) and 1445 (C=C), 1109, 856.

HRMS m/z 715.2142 [M+HCOO]$^{-}$ cal. 715.2142

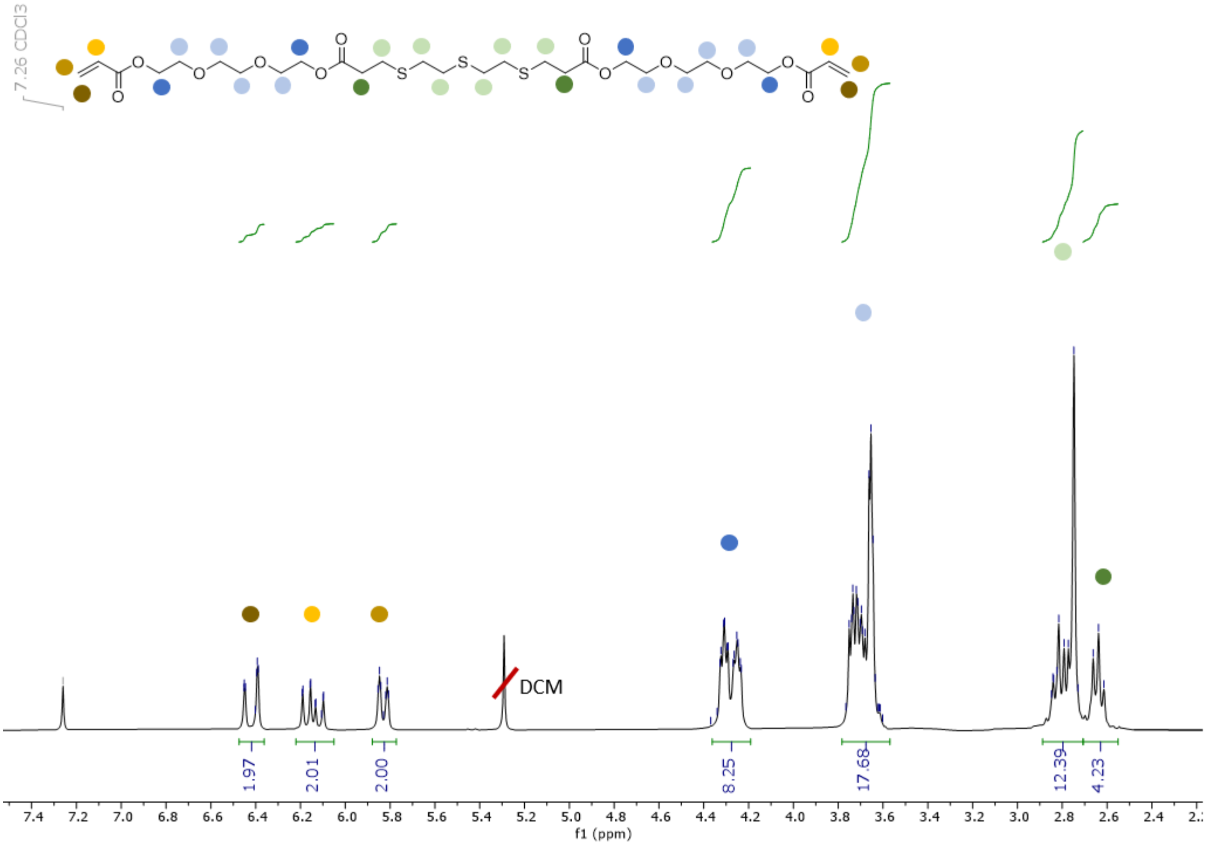

Figure S3. ^{1}H NMR spectrum of EG_3SA in $CDCl_3$.

^{13}C NMR (75 MHz, $CDCl_3$) δ 171.88, 166.26, 131.17, 128.39, 70.86, 70.68, 69.25, 64,09, 63.95, 63,74, 35.39, 34.90, 32,50, 32,38, 32.30, 29.80, 27.21, 27,13.

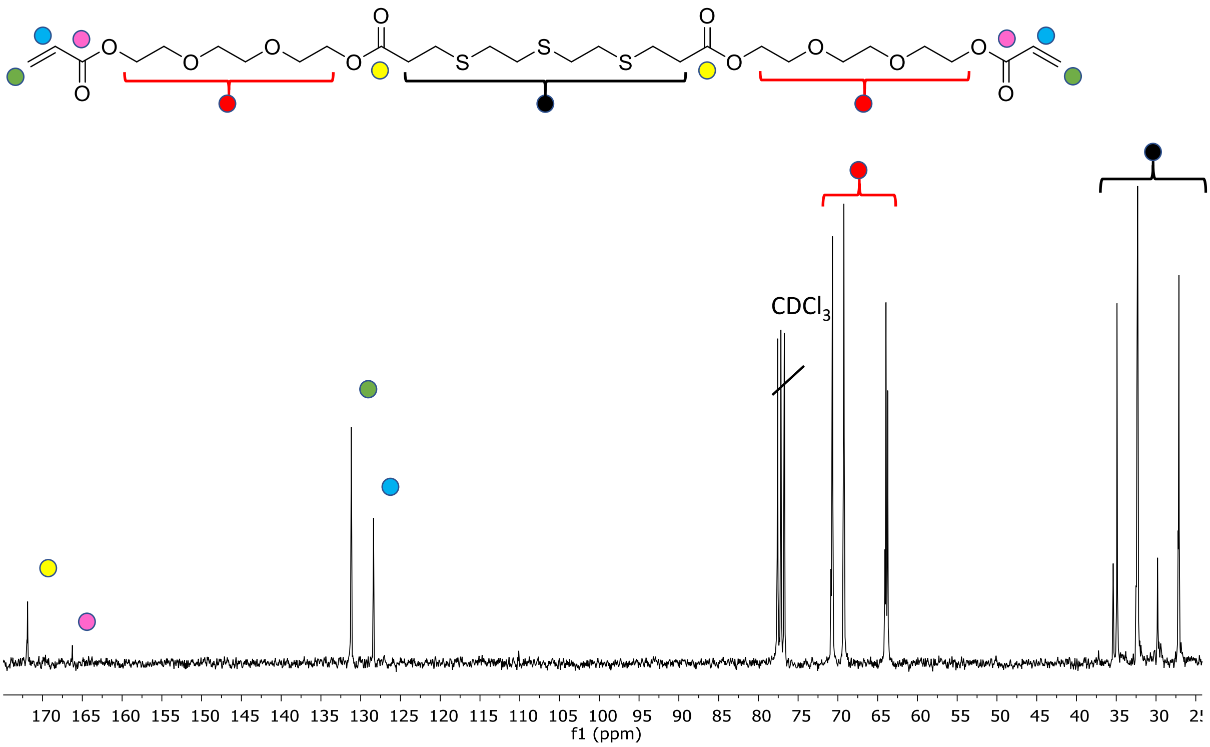

Figure S4. ^{13}C NMR spectrum of EG_3SA in $CDCl_3$.

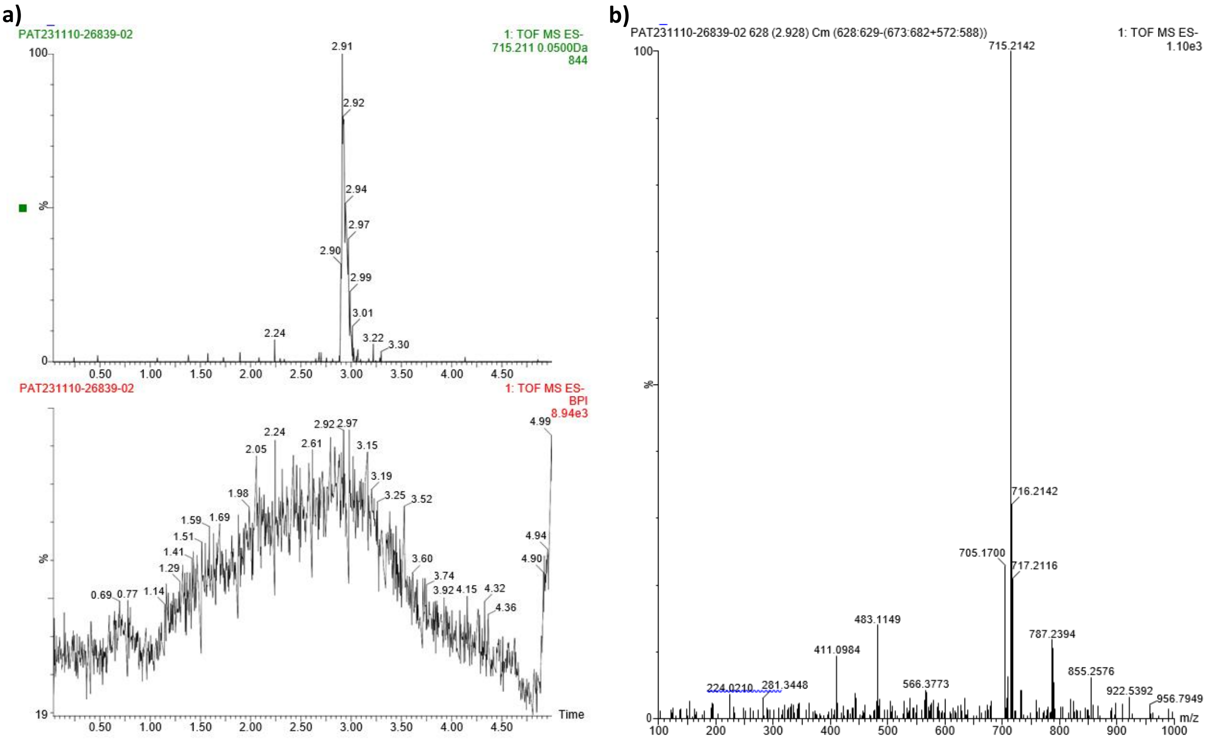

Figure S5. UPLC mass spectrometry of EG_3SA.

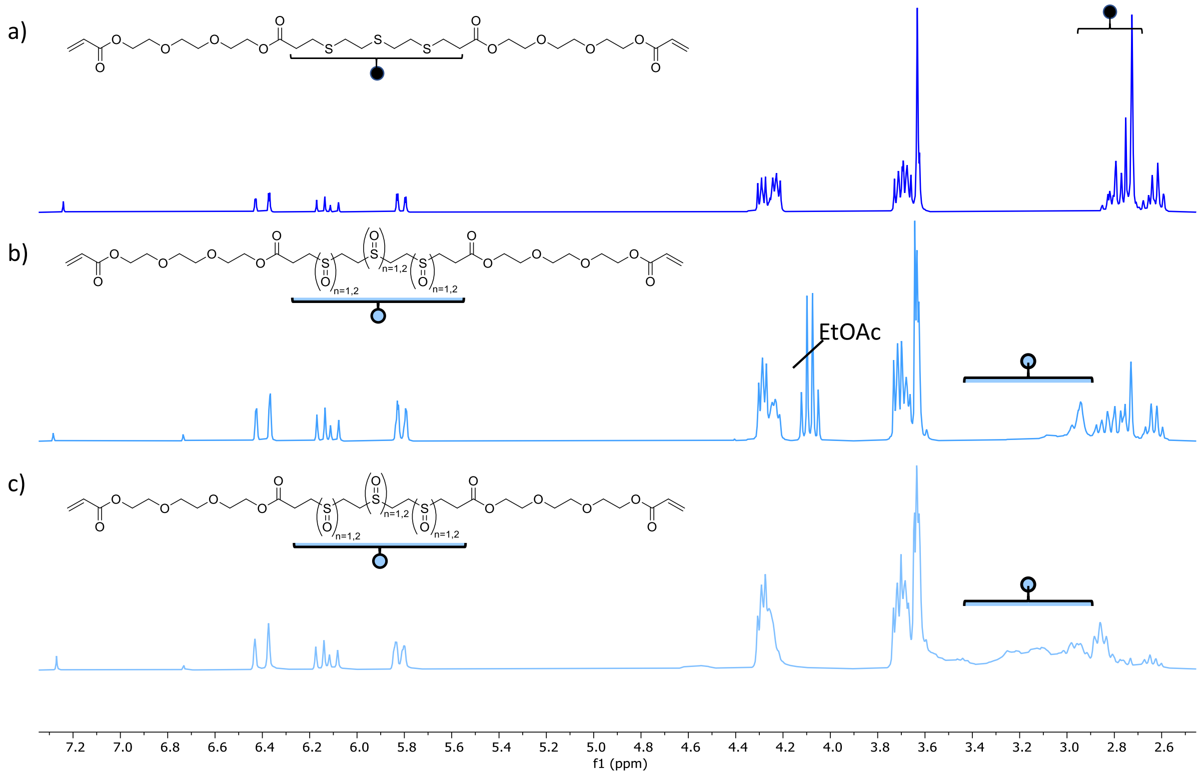

Figure S6. a) ^{1}H NMR in $CDCl_3$ of the starting monomer, b) ^{1}H NMR in $CDCl_3$ monitoring of the oxidate reaction 2 h, c) ^{1}H NMR in $CDCl_3$ of the oxidized product after 4 h.

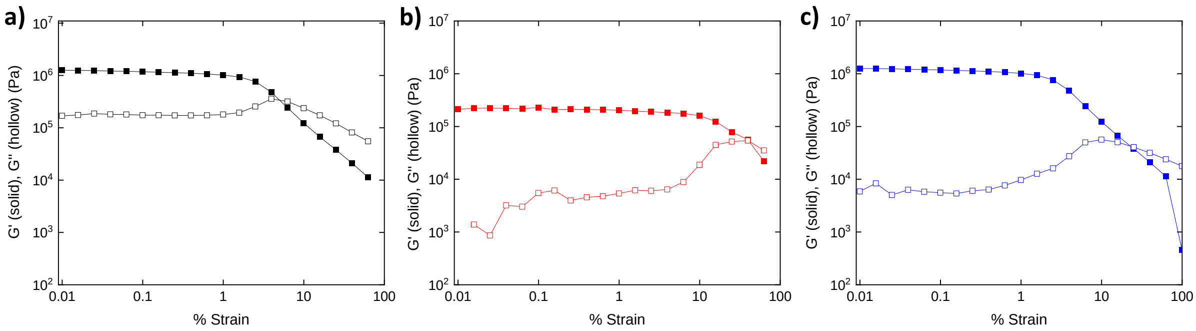

Figure S7. Strain sweeps at 1 Hz frequency of (a) PEG_1SA, (b) PEG_2SA, and (c) PEG_3SA hydrogels swollen in PBS. (d) Frequency sweeps at 1% strain of PEG_nSA (n = 1, 2, 3) hydrogels swollen in PBS.

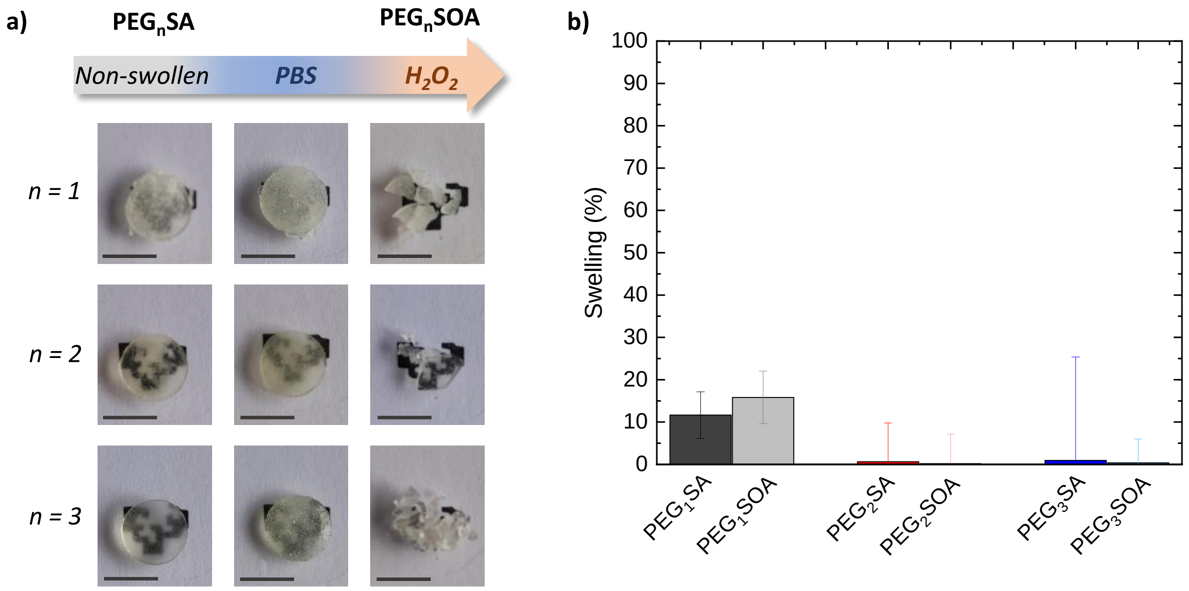

Figure S8. (a) Pictures of the swollen PEG_nSA (n = 1, 2, 3) hydrogels in PBS (pH 7.4) under non-oxidative conditions and in the presence of 9 mM H_2O_2. Scale bars = 5 mm. (b) Swelling evolution of the non-oxidized (PEG_nSA) and oxidized (PEG_nSOA) hydrogels.

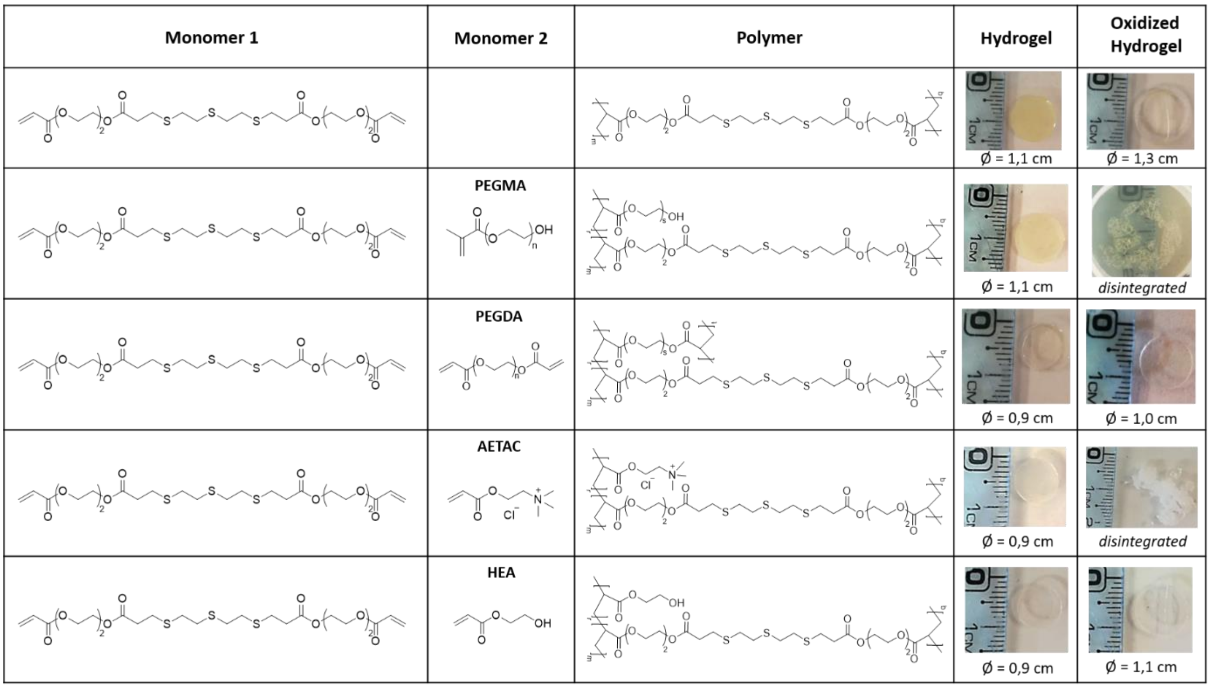

Figure S9. Photo-polymerization of EG_2SA together with different monomers leading to hydrogels, with the pictures of those hydrogels before and after oxidation in the presence of H_2O_2.

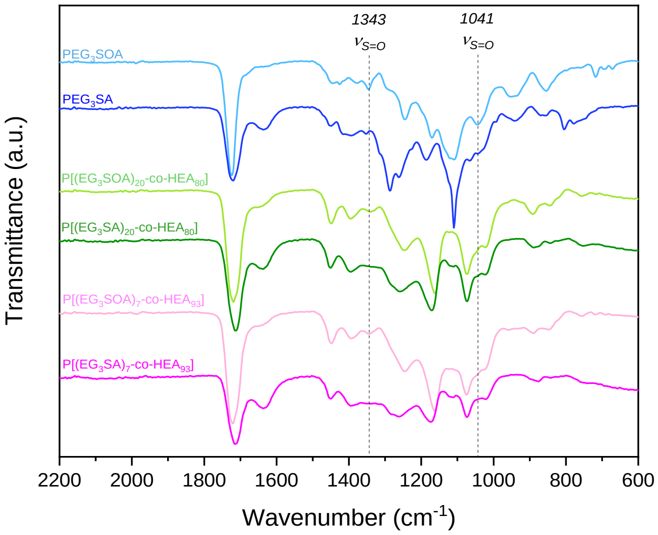

Figure S10. FTIR spectra of PEG_3SA and P[$(EG_3SA)_x$-co-HEA_y] hydrogels before and after oxidation in the presence of H_2O_2 for 4 h.

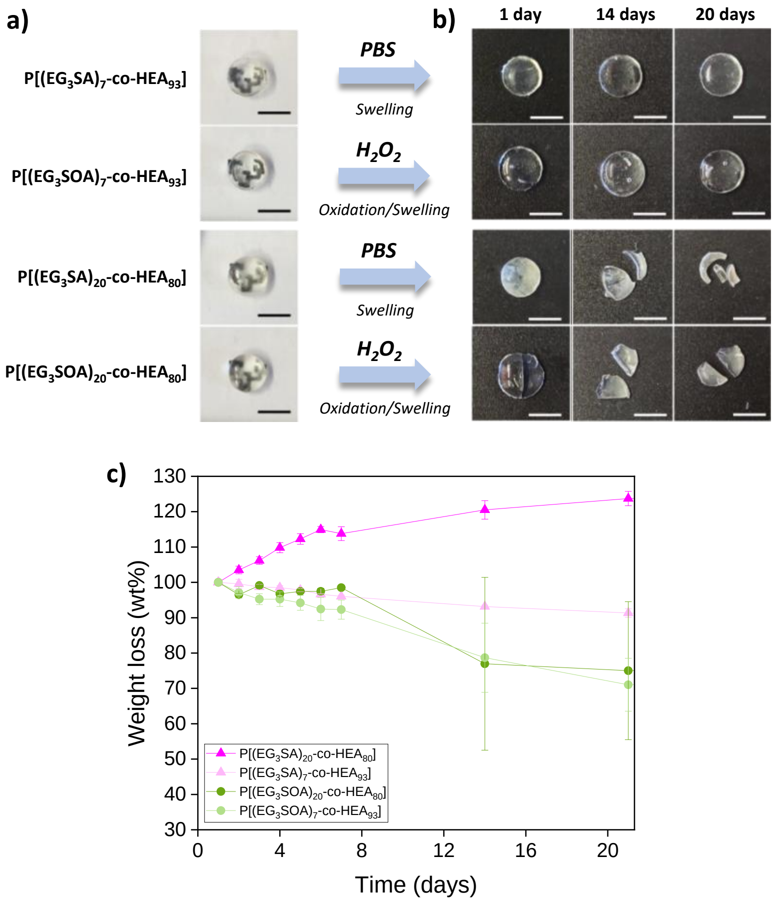

Figure S11. Pictures of P[$(EG_3SA)_x$-co-HEA_y] hydrogels a) before and b) after immersion in PBS (pH 7.4) or 9 mM H_2O_2 for 21 days to determine their degradation properties. Scale bars = 5 mm. c) Weight loss of the hydrogels over degradation time.

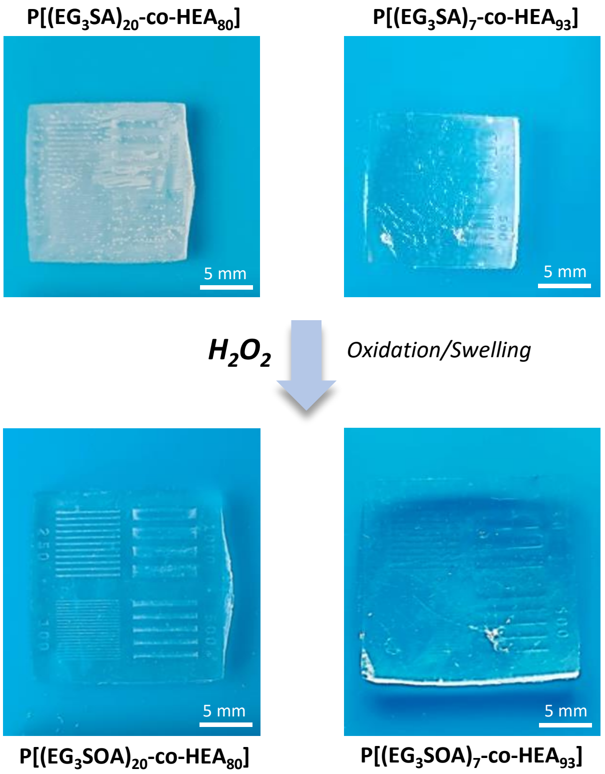

Figure S12. DLP resolution tests of shape-defined 4D printed P[$(EG_3SA)_x$-co-HEA_y] hydrogel scaffolds with lined holes of variable line widths, before (top) and after swelling in the presence of 9 mM H_2O_2 (bottom).

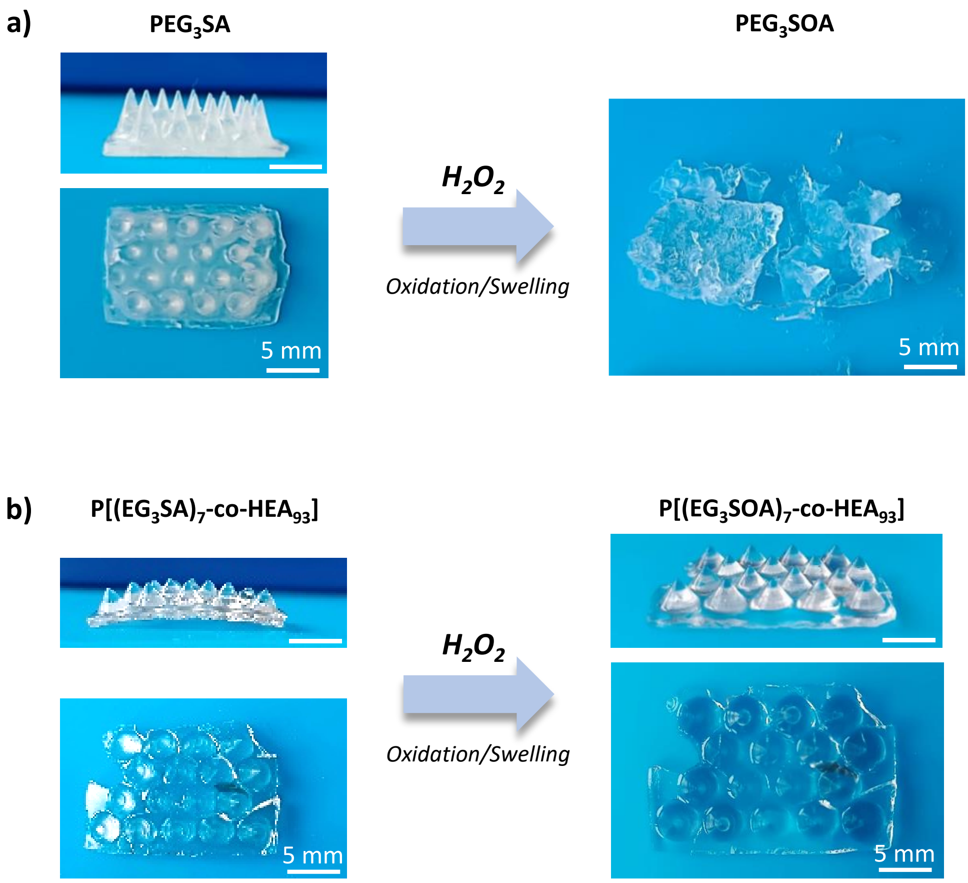

Figure S13. Shape-defined digital light 4D printed (a) PEG_3SA and (b) $P[(EG_3SA)_7$-co-$HEA_{93}]$ hydrogels, before (left) and after swelling in the presence of 9 mM H_2O_2 (right). Scale bars = 5 mm.
